# Understanding the Professional Care Experience of Patients with Stroke: A Qualitative Study Using In-Depth Interviews

**DOI:** 10.5334/ijic.6526

**Published:** 2022-10-07

**Authors:** Belen Martin-Sanz, Rosa María Salazar-de-la-Guerra, Juan Nicolas Cuenca-Zaldivar, Ana María Aguila-Maturana, Cristina Garcia-Bravo, María Salcedo-Perez-Juana, Ester Capio-Calatayud, Domingo Palacios-Ceña

**Affiliations:** 1 Research Group of Humanities and Qualitative Research in Health Science of Universidad Rey Juan Carlos (Hum&QRinHS), ES; 2 Servicio Madrileño de Salud (SERMAS), ES; 3 Hospital Universitario Fundación Alcorcón, ES; 4 Department of Physical Therapy. Centro de Hospitalización y Rehabilitación, ES

**Keywords:** (MeSH): stroke, professional-patient relations, therapeutic alliance, stroke rehabilitation, integrated care, qualitative research

## Abstract

**Background:**

Professional support and communication stimulates the professional-patient relationship and supports the recovery of stroke patients.

**Objectives:**

To describe the perspectives of patients with stroke regarding communication, professional support, and their ability to participate in processes and integrated care with health providers.

**Methods:**

A qualitative study was conducted. A purposeful sampling and snowball-technique were used. Patients diagnosed with moderate or severe stroke in the post-acute or chronic stage of the disease were included. Data collection consisted of in-depth interviews and researcher field notes. A thematic analysis was performed.

**Results:**

Thirty-one patients were included. Three themes were identified: 1) Providing support, with four categories, professional behavior, personalized attention, the heart of the professional and building a bond with the patient; 2) Facilitating communication, with three categories, the patient as the recipient, the content of the message and the channel, and the professional as the person that conveys the message; and 3) Promoting participation, with two categories, barriers, and incentives to participate.

**Conclusions:**

When providing support, professionals should consider communicating information and encouraging the participation of stroke patients for integrated care.

## Introduction

Stroke is the third leading cause of death worldwide and is a major cause of disability [1]. In Spain, according to the report by the Spanish Society of Neurology, entitled “the Atlas of stroke Spain 2019” [2] about 110,000 people suffer a stroke each year, of whom at least 15 percent will die and, among the survivors, 30 percent will present disability and functional dependence. This report [2] predicts that these figures will continue to increase by 39% between 2015 and 2035. The quality of care of people with stroke must be guaranteed, and greater clinical effectiveness and patient satisfaction must be achieved for integrated care [3,4]. Integrated care of the stroke patient should be based on collaborative engagement and cooperation between patients and caregivers to align health care services resulting in effective clinical stroke care management [5]. It is necessary to favor the implementation of this integrated care in stroke patients by facilitating the management of patient preferences in the care process [6], requiring rigorous evaluation in this population through training and patient empowerment of their health care [7]. The patient’s perspective on their health care affects adherence to treatment or the reduction of complaints and is considered a proxy indicator of care [8].

The relationship between professionals and patients is considered a relevant element in the quality of health services [3,4]. The professional-patient relationship is defined as the set of guidelines and behaviors that are established in the clinical encounter, as well as the significant therapeutic interactions shared with patients and healthcare professionals [9]. This type of relationship is valued above technical skills and can influence a healthcare professional’s recommendations [10]. In addition, patients positively value the existence of the professional’s interpersonal care. This care consists of treating patients, including affective behaviors, and communication management skills, and facilitating patient participation in decisions [11].

Within the professional-patient relationship, professional support strengthens the relationship and increases patient satisfaction [4,11]. This support can take different forms such as professional kindness, warmth, active listening, consolation, forgiveness, or acceptance of behaviors [4]. In addition, the assessment of how the patient wants to be treated according to their disease, culture, ethnicity, age, etc., is also included [12]. Moreover, communication is another element in the professional-patient relationship [13], which facilitates treatment efficacy [14] and improves patient satisfaction [15]. In the case of stroke patients, communication between trained professionals and patients promotes shared decision making and prevents ethical conflicts [16]. Finally, the participation of patients facilitates their adherence to treatment, improves the perceived quality of care, and facilitates shared decision making with professionals [17].

It is necessary to explore what stroke patients expect from the support of health professionals, what it means for them to receive professional support, how the communication process is carried out, and how decision making between the professional and the patient is carried out. In this manner, essential information may be obtained for professionals to use in their daily clinical work of integrated care with stroke patients. Thus, the objective of this study was: to describe the perspectives of patients with stroke regarding the communication, professional support, and participation of patients with health providers.

## Methods

### Study Design

A qualitative descriptive study was conducted [18]. The aim of a descriptive qualitative study is to identify an event and describe “what is happening” and “how it is happening” [19,20]. Qualitative descriptive studies aim to be a comprehensive summary of events in the everyday terms of the described event [21,22]. Qualitative research is useful for describing complex phenomena and understanding the beliefs, values, and motivations that underlie individual health behaviors [18,23]. Furthermore, qualitative studies have been used to research the stroke survivors’ experiences and expectations before and after treatment [24], and the patient’s participation in their recovery process [25]. The Standards for Reporting Qualitative Research (SRQR) were used [26].

### Theoretical Framework

The theoretical framework that guided this study was interpretivist [18]. From an interpretive perspective, human action is meaningful, and the goal of inquiry is understanding how people respond and understand the meaning of social phenomena [27].

### Research team

Eight researchers were involved in this study (six women, two men), of which four had experience in qualitative designs (DPC, BMS, CGB, MSPJ). Three hold PhDs in health sciences (AMAM, DPC, JNCZ), and were not involved in clinical activity. Three members of the research team worked in the rehabilitation context (BMS, AMAM, ECC).

### Setting and Sample

A non-probabilistic, purposeful sampling and snowball-technique strategy were used in the present study, based on relevance to the research question rather than representativeness [18,28]. A purposeful sampling strategy involved deliberately selecting participants [18]. Also, a snowball sampling procedure was applied, in the case of participants who put the researcher in touch with other participants in similar circumstances and who met the inclusion criteria.

The inclusion criteria were: a) patients > 18 years, b) with moderate or severe stroke diagnosed by a doctor according to the National Institutes of Health Stroke Scale (NIHSS) [29], c) in the post-acute or chronic stage. The exclusion criteria were: a) patients with cognitive decline, and/or with alterations in verbal communication, b) patients with mild stroke according to the NIHSS Scale, and c) in the acute stage. Participants were recruited from two stroke rehabilitations centers.

In qualitative research, there is no formula for the prior calculation of the sample size, since the results are not intended to be representative and generalizable [18,28]. In the current study, the sample size was determined following the proposal by Turner-Bowker et al [30]. These authors reported that 99.3% of concepts, themes, and contents emerged with around 30–35 interviews [30]. With this proposal, a greater capacity to identify codes, categories, and topics is achieved.

### Data Collection

In-depth interviews and researcher’s field notes were used as the main tool for data collection [18,28]. With participants 1–5, the interview started with an open question: “Please, can you share your personal experience with me regarding stroke and the communication process, professional support and patient participation during your rehabilitation and recovery process?” Thereafter, the researchers noted the key words and topics identified in the patients’ responses and used their answers to ask for them to clarify the content [18,28]. A first analysis was performed on the unstructured interviews of participants 1–5. This analysis revealed some relevant topics that required further study, thus making it necessary to include a second stage of data collection. The second stage (participants 6–31) consisted of semi-structured interviews that were based on a question guide designed to gather information regarding specific topics of interest (Table 1). The semi-structured question guide did not follow a fixed order of questions. The interviewer and the participants could start or continue with different questions that probed different areas of research.

**Table 1:** Semi-structured interview guide.


RESEARCH AREAS	QUESTIONS

Health care	How was the health care? Where did you go for help? What was the most relevant aspect of the health care you received? How was the rehabilitation during your stay in the hospital? And after discharge from the hospital? Why and in what aspects?

Role of the health professional	In your opinion, what is the role of the involvement and attitude of the health professional in your rehabilitation process? What aspects make a professional relevant to you?

Professional-patient relationship	What relevance does the professional-patient relationship have for you? What is most necessary on behalf of the professional? and on behalf of the patient? What is most relevant for you in this relationship? What role does communication play in the professional-patient relationship? What should it be like for you? What about professional support? What about your participation in your process?

Barriers and facilitators in the therapeutic relationship	What barriers and/or facilitators can influence the therapeutic relationship? What about communication? What about professional support? What about your participation during your process?


The interviews were audio-recorded and transcribed verbatim. Overall, 1273 min of data collection were recorded, with a mean of 41 min (SD 15.8). All interviews were held at stroke rehabilitations centers. We used researcher field notes as a secondary source of information to provide more in-depth information [28]. In addition, field notes provide a rich source of information as participants describe their personal experiences and their behaviors during data collection.

### Data Analysis

**Figure 1:**
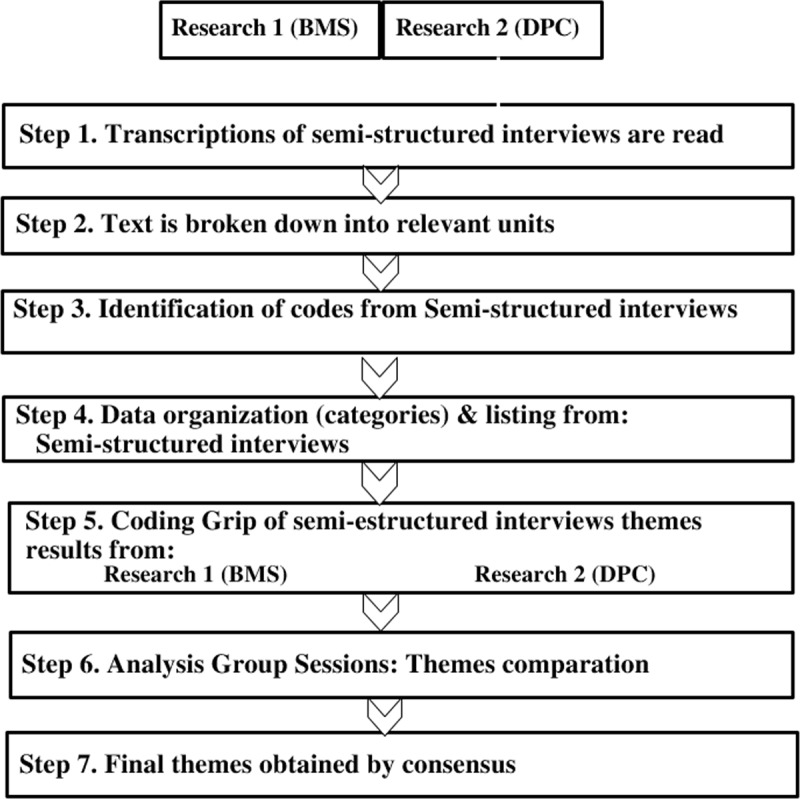
Description of the data analysis process.

An inductive thematic analysis was used on the interviews to identify the relevant themes obtained from the interviews [28,31]. Full transcripts were made of each in-depth interview and of the researchers’ field notes [28,31]. Thematic analysis consisted of identifying text fragments with relevant information to answer the research question [28,31]. From these narratives, the most descriptive contents (codes) were identified. Subsequently, these units were grouped by their common meaning (categories) and/or similar content [28,31]. Thematic analysis was applied separately to interviews and field notes by BMS, and DPC. Joint team meetings were held to combine the results of the analysis and discuss data collection and analysis procedures. In these team meetings the final themes were displayed, combined, integrated, and identified. In case of divergence of opinions, the identification of the theme was based on consensus among the members of the research team. See Figure 1.

### Methodological Rigor

We used criteria by Guba and Lincoln for establishing trustworthiness of the data by reviewing issues concerning data credibility, transferability, dependability, and confirmability [32]. Table 2 summarizes the procedures used to enhance trustworthiness.

**Table 2:** Trustworthiness techniques.


CRITERIA	TECHNIQUES PERFORMED AND APPLICATION PROCEDURES

Credibility	Investigator triangulation: each interview was analyzed by two researchers. Thereafter, team meetings were performed in which the analyses were compared, and themes were identified. Triangulation of data collection methods: unstructured, semi-structured interviews were conducted, and researcher field notes were kept. Member checking: this consisted of asking the participants to confirm the data obtained during the data collection.

Transferability	In-depth descriptions of the study were performed, providing details of the characteristics of researchers, participants, contexts, sampling strategies, and the data collection and analysis procedures.

Dependability	Audit by an external researcher: an external researcher assessed the study research protocol, focusing on aspects concerning the methods applied and study design.

Confirmability	Investigator triangulation, data collection and analysis triangulation. Researcher reflexivity was encouraged via the completion of reflexive reports and by describing the rationale for the study.


### Ethics

The current study was approved by the Ethical Committee of Universidad Rey Juan Carlos (code: 2106201911119) and the Ethical Committee of Hospital Universitario Fundación Alcorcón (code: 19/69). Participants provided oral informed consent prior to their inclusion in the study.

## Results

The sample consisted of 31 patients with stroke (11 women) with a mean age of 64 years (SD = 15) with a NIHSS scale of 64.52% (n = 20), and 35.48% (n = 11), indicating moderate and severe stroke, respectively. The characteristics of the participants are shown in Table 3.

**Table 3:** Profile of participants.


NUMBER OF PARTICIPANTS	N = 31

Sex	Female: n = 11 (35.48%) Male: n = 20 (64.51%)

Age	Average: 64 years SD: 15

Stroke type	Hemorrhagic stroke: n = 7 (22.58%) Ischemic stroke: n = 24 (77.42%)

Time of stroke evolution	Average: 38 months SD: 35

NIHSS scale	Moderate: n = 20 (64.52%) Severe: n = 11 (35.48%)

Barthel index (functional state)	Mild: n = 11 (35.48%) Moderate: n = 12 (38.71%) Severe: n = 8 (25.81%)


### Results of the Thematic Analysis

The themes that explain the professional care experience of patients with stroke were 1) Providing support, with four categories, professional behavior, personalized care, the heart of the professional and building a bond with the patient; 2) Facilitating communication, with three categories, the patient as the recipient, the content of the message and the channel, and the professional as the conveyer of the message; and 3) Promoting participation, with two categories, barriers, and incentives to participate. See Table 4, Identified themes and categories.

**Table 4:** Themes and categories.


THEMES	CATEGORIES

Theme 1. Providing support	Professional behavior Personalized care The heart of the professional Building a bond with the patient

Theme 2. Facilitating communication	The patient as the recipient The content of the message and the channel The professional as the conveyer of the message

Theme 3. Promoting participation	Barriers to participation Incentives to participate


Participants’ narratives, extracted directly from interviews, described each identified theme [26]. A detailed summary of the categories, and narratives which justified the themes obtained is shown in Supplementary File, Table S1. Summary of the themes, categories, and narratives.

#### Theme 1: Providing support

This theme describes how the professional can support the patient, according to their behavior, and provide personalized care, where the professional builds a bond with the patient.

Category: Behavior of the professional.

Patients highly valued professionals paying full attention during their consultation or treatment, without simultaneously performing other tasks (i.e., checking their phone, typing on the computer). Furthermore, “the fact that the patient talks and the professional listens” was associated with being a good professional and was seen as a stimulus to share their experiences and provide information. When the professional asks the patient questions, this was perceived as a sign of concern on behalf of the professional: “*It seems very important to me, to be asked how you are doing with your disease. It shows interest and concern and provides an indication of the type of professional you are.” (P17)*. This means that they are paying attention to the person’s problems and seeking to understand them. Patients considered that the fact that the professional displays commitment to solve their problems, and professional dedication, and has gestures with patients that make them feel comfortable and safe, are all aspects that show that they are with a high-quality professional: “*It shows in the shifts. We can all do a job, but the attitude with which things are done is relevant. Maybe things don’t work out, but you put all your love and all your effort into it, so, for me that’s enough.” (P2)*

Category: Personalized care.

For patients, personalized care means knowing and understanding their needs, their family context, their way of life and their emotions, and adapting the treatment as necessary. In addition, the professional must consider patients’ concerns, the importance of the disease in the patient’s life, the role of values and beliefs, and assign value to their emotions and feelings: “*I have concerns, I’m sure they won’t solve all of them, but it is important to perceive that they are concerned about solving the most important ones for me.” (P29)*. Patients described how the disease can be experienced differently by each person, which conditions their priorities, demands and expectations to solve it.

The patients interviewed pointed out that ’t is necessary for professionals to share time and space with patients, thus, professionals should not be in a hurry, they should try to face work overload, avoiding actions becoming automatic and should endeavor to ensure that care is not impersonal or an administrative formality. Patients noted that the extent of the professional’s interest is determined based on the time dedicated to the patient.

Category: The heart of the professional.

The patients who participated in this study described how the way they are treated influences how they cope with the disease and their acceptance of the treatment. Thus, when they perceive “affection” from the professional, described by patients as “that spark” that makes them feel better, they feel that the professional is approachable and therefore not cold and distant: “*The spark is giving a lot of love and affection (…) When you put your affection into something, everything feels better. It’s something you notice in people, and it makes a difference among professionals.” (P2)* Some patients referred to this as receiving “caring” treatment and feeling “pampered”. Another key aspect was “closeness”. Patients described that “a close professional” is one who supports the patient, who is a reference figure, and can be counted on. Patients described feeling “swaddled”. The narratives describe how physical contact with the professional provides them a sense of security. Shaking hands, a hug, a comforting caress, conveys companionship, and reminds them that they are not alone: “*Do you know what it’s like to have someone give you a hug in this situation? It’s what you need at that moment, a help you don’t expect, the gesture that you’re not alone.” (P29)* Finally, patients described that smiling at the patient is perceived as a sign of closeness and companionship; they felt that the professional conveys joy.

Category: Building a bond with the patient.

The patients interviewed noted the importance of getting to know personal aspects of the professional, and for the professional to also open up to them. This allowed them to experience a more personal relationship with the professionals, beyond the provision of treatment or care. They already knew the professional, and now they know the person. For patients, this means the professional is placed on the same level of mutual knowledge as themselves. Consequently, patients described that the treatment changes, they have more confidence, they can ask questions, and express themselves without filters or fear.

Patients emphasized how “putting themselves in the other person’s shoes”, “getting into their skin” would help professionals to understand them better, increase their trust and improve the professional-patient relationship: “*It seems like an everyday thing that doesn’t require any effort, however, for me it was a whole new world. It is necessary for a professional to understand what a stroke means and the consequences it has, from the patient’s point of view (…) If they do, when I say I’m tired, they understand that I’m tired, because they are putting themselves in my place and know what is happening to me. That gives me confidence. Respecting others is much easier, when you put yourself in their place, and by understanding what happens to patients, everything is much easier.” (P20)* For patients, trust is everything, however, it is not given freely, it must be earned. Thanks to trust, the patients put themselves “in the professional’s hands”. Mistrust appears when the professional loses credibility. This appears when they feel deceived, when a treatment is prolonged longer than expected (entailing costs for the patient), when a professional fails to recognize their mistakes, or to show interest, and when they are unfamiliar with the disease and/or its evolution. Finally, humor is considered as a facilitator of the relationship with the professional. Sharing moments of humor and laughter enables the patient to relax and enhances the feeling of closeness with the professional. Humor is displayed by making jokes with professionals. For patients, a feeling of camaraderie and complicity is generated: “*It’s essential. If you laugh with someone then it’ s different (…) There is a camaraderie. And when you go to therapy, you make jokes, they encourage you, and the treatment is experienced differently.” (P6)*

#### Theme 2: Facilitating communication

This topic describes how communication should be from the patients’ perspective and which elements facilitate or hinder it.

Category: The patient as the recipient.

Patients appreciated it when the professional adapted the message and the information they want to transmit, avoiding technical language. The use of technicality was perceived as remoteness or coldness on behalf of the professional: “*You can tell when there is no closeness with the professional, because they only use technical terms, such as ischemic damage. And when you ask him what it is, he responds with more words that you don’t understand.” (P6)*. When the message was tailored to the patient, patients felt that they were “talking on the same level” with the professional, even if it was not the same vocabulary. Patients reported how, at times, professionals chooe to convey the information to another person first, usually a family member. This was a source of anger and frustration among patients, since they prefer to be the first ones to receive the information, regardless of the reasons for informing the family first, for example, to avoid worrying the patient. According to the patients interviewed, the information received tends to be accepted without being questioned, in cases where there is a previous relationship, or the professional knew the patient.

Category: The content of the message and the channel.

The patients emphasized that they need to receive information about their disease, its causes, implications, and evolution. They require information that will help them to understand how the disease will impact their life and that of their family. Any information that does not meet these criteria is considered useless. Moreover, patients reported that some professionals include words of encouragement and support in the technical information contained in their message. This is experienced as a “morale booster” and is seen as a sign of the professional’s concern and involvement: “*When it comes to stroke, the professional must be positive, to help you, to encourage you. Saying a positive word to you doesn’t hurt. By encouraging me, he showed me that he understood perfectly well what I had, and I was trying hard to get better.” (P17)* Patients also pointed out the need to establish official communication channels, as well as the use of alternative communication channels such as telephone and e-mail. However, alternative channels are avoided because they lose the proximity with the professional.

Category: The professional as the conveyer of the message.

Patients recounted that health professionals should be trained to convey information and develop skills to improve their communication with patients. The technical knowledge of the profession is different from the communication skills they must develop. This training should be mandatory for all professionals. Communicating while avoiding bluntness with patients is perceived positively and is highly desirable: *“It’s not just about explaining things, you must know how to explain it. When you have a certain responsibility, you must know how to communicate. You have to give feedback in a non-aggressive way, even if it is negative feedback. You have to know how to give negative news without crushing anyone.”(P29)*. In addition, receiving certain information or the way it is conveyed is seen as an obstacle to recovery. Patients described that some professionals only focus on “what has been lost”, on the limitations that remain, on the sequelae, on what is not going to be recovered. In addition, they focus all their information on informing the patient of the limited possibility of rehabilitation, on what they will not be able to do. This is experienced as “dynamiting” the patient’s recovery and they feel “marked” by the professional’s words.

#### Theme 3: Promoting participation

This theme describes barriers and facilitators for patient participation.

Category: Barriers to participation.

Some patients reported that they preferred not to give their opinion or participate in their process or in the planning of activities, since they lack knowledge. They tended to listen to what the professional said. Other patients felt that they were unable to give their opinion or act, as they were not involved in the decisions. They saw themselves as “the last link” in the chain. Treatment objectives are set without the patient’s input and participation. The patient must accept the objectives and treatments that are prescribed and wait to see if they work. “To accept or to wait”, is seen by patients as a way of not involving them in their process. Patients narrated that some professionals try to act as if they know what it is like to live with the disease, more so than the patient who is suffering from it. Patients described feeling a barrier when the professional acts pretentious. The professional knows everything by virtue of being a professional: “*The roles are very different. There is an invisible barrier that you notice right away; those who are in charge and those who are not in charge, those who know and those who don’t know.” (P10)*. Consequently, patients perceived a lack of control, as they cannot decide, they lose control and feel like they are in someone else’s hands.

Category: Incentives to participate.

The professional actions that facilitate patient participation include asking about their process, providing information on therapeutic decisions and changes, continuous updating on the evolution of their disease and allowing each patient to participate in their recovery according to their possibilities. In addition, patients reported that being consulted about their preferences encourages them to participate in the shared decision-making process: “*There are two different approaches used by health care providers. On the one hand, those who don’t allow you to participate and tell you what to do, and on the other, those who consult you. I prefer the people who try to get you more involved. It means that they ask me what I expect, how I want to do it. Considering me as a person who can contribute things. It means talking together to see how we can do it.” (P9)* For them, the recovery process is a joint process between the patient and the professionals. Sometimes, patients even described that they needed to feel the involvement and desire of the professionals to get them back on the “playing field” and participating again. Furthermore, the fact that the professional corrects the patient’s mistakes or errors and redirects them during treatment is well received and does not prevent the patient’s participation in decisions. This is appreciated as a sign of the professional’s involvement.

A summary of the facilitators (enablers) and barriers of professional support, communication and participation with stroke patients is shown in Supplementary File, Table S2.

## Discussion

Our results revealed the elements that professionals can use to support and build bonds with patients, improve communication, and facilitate participation from the perspective of stroke patients. Patient experience is more than “patient satisfaction,” and asking patients “what happened” during an episode of care is more valid for judging the quality of care than simply asking about “satisfaction” [3,33]. The National Institute for Health and Care Excellence [34], developed a quality standard that provided healthcare professionals with clear guidance on the components of a good patient experience, summarized in six quality statements against which patient experience can be measured. These statements include empathy, dignity, and respect; contacts for ongoing care; information exchange; individualized care; preferences for sharing information; and decision making.

Regarding the aspects that influence the patient’s perspective on the support and professional help received, previous studies described how patients have expectations of building a relationship based on caring, and where they expect professional behaviors and attitudes to match or respect their needs [35]. Pallesen et al. [36] and Batbaatar et al. [4] showed how patients consider and perceive the support perceived by professionals as a cornerstone that influences their recovery process. Professional support influences the satisfaction perceived by stroke patients and their families and should be based on the individual characteristics of each patient and family [37]. The professionals’ consideration of the expectations of the stroke patient and their family, considering their particularities, together with the professional’s efforts to improve the quality of interpersonal care and treatment of the patient, facilitate the patient-professional relationship [36]. Considering the patient’s individuality, personalized care means knowing the patient’s needs, concerns, expectations and emotions. This forces the professional to adopt different roles in the relationship with the patient. Parker et al. [11] showed how patients expect physicians to act as a drug (the relationship with the physician is therapeutic for the patient), as a detective and validator (identification and categorization of symptoms), and as a collaborator (patients prefer a collaborative partnership). Also, according to Parker et al. [11] patients are immersed in mental and emotional concerns (stress, anxiety, fear, uncertainty) that can affect their recovery. Professional support, through close accompaniment (based on the patients’ needs and context), helps stroke patients to understand and validate their concerns, and helps to build a trusting relationship based on empathy, care and understanding [38]. This improves the bond and the relationship between the professional and the patient and improves the patients’ sense of security by reducing post-stroke stress and anxiety [39]. Previous studies [24,40] reported how patient-centered care is an element of support for the person who has suffered a stroke, and of relevance in clinical management, which should be considered as another element in the recovery and treatment planning process.

Communication is essential in the experience of stroke patients and is the key tool for the professional to provide clear and accessible information to patients [33]. To facilitate and improve communication, the professional must consider the patient as the receiver of the information, adapting the message, speaking at the same level, and trying to avoid the use of highly technical language [34]. Previous studies describe how employing strategies to promote communication and understanding avoids disappointment and unrealistic goals in stroke patients [41]. In addition, the professional must know how to convey the information, providing information about the disease, and conveying words of encouragement in the message [41]. In the systematic review by Burton et al. [42] regarding the experiences of patients with acquired neurological conditions and their caregivers about the process of receiving information about recovery, the importance of providing the right information at the right time is described, together with managing expectations and the way in which the message is given, learning to talk about recovery, and managing emotions in an uncertain context such as the recovery of acquired neurological processes. Communication between the stroke patient and healthcare professionals helps to adjust patients’ expectations towards recovery during rehabilitation [43]. In addition, professionals should avoid highlighting the limitations of patients who have suffered a stroke [44]. This inadequacy of the information provided by professionals generates anxiety and fear and discourages participation in recovery [44].

In relation to communication, patient participation is based on shared understanding and comprehension between patients and professionals in order to make decisions about the short and long term recovery process of stroke patients [24]. Our results reveal how patients encounter barriers to participation, considering that their process is in the hands of others. Previous studies described how stroke patients admitted to specialized rehabilitation centers did not participate in any aspect of post-acute care [45]. Some of the reasons for this include lack of knowledge or lack of skills to re-engage [46].

Lindblom et al. [25] described how patient involvement should be based on professional-patient interaction and clear assignment of roles. Previous studies [47] highlight different professional factors that facilitate the participation of stroke patients, such as consulting them about their expectations, showing involvement in the patient’s recovery, and jointly establishing therapeutic objectives and goals [25]. Moreover, Parsons et al. [48] in their research on stroke rehabilitation clinician’s perceptions of the patient as an active partner in setting goals within stroke rehabilitation and factors that influence patient engagement, identified barriers to the participation of patients and their families such as knowledge of the patient and family, the role of the patient in setting goals, the effect of clinician’s attributes on goal setting, goal-setting at the home versus hospital, and professional/funder expectations of clinicians.

The present study has several limitations. First, one of the researchers had contact with the participants. However, data collection and analysis were controlled by other members of the research team. Secondly, participants were asked about “health professionals” without specifically focusing on any type of professional. This was done to give participants more freedom to respond and to avoid directing or limiting their response.

## Conclusions

Based on the perspective of our participants, our results show relevant points that professionals should consider when providing support, communicating information, and encouraging the participation of stroke patients. In addition, barriers and facilitators are identified for providing support to patients, for communication and transmission of information, and for encouraging the participation of stroke patients in their recovery.

Considering the stroke patient’s perspective on how the professional should support, communicate, and provide information, and encouraging patient participation has great clinical relevance for health professionals, as it can enhance the professional-patient relationship and impact decision making, treatment regimens, and adherence to health care recommendations and interventions. This improves the holistic understanding of the stroke patient in integrated care, increasing the quality of care and its results.

## Additional File

The additional file for this article can be found as follows:

10.5334/ijic.6526.s1 Supplementary files. Table S1 and Table S2.
