# Supplementary material for: Understanding the Professional Care Experience of Patients with Stroke: A Qualitative Study Using In-Depth Interviews
Source: Int J Integr Care. 2022 Oct 7;22(4):2. doi: 10.5334/ijic.6526 (PMC9541171; doi:10.5334/ijic.6526)
Supplement: Supplementary files. — Table S1 and Table S2. [file ijic-22-4-6526-s1.pdf]

Table S1. Summary of the themes, categories, and narratives.

Theme 1: Providing support	
Categories and definition	Narratives
Category: Behavior of the professional<br>Describes those actions and behaviors of professionals that are positively valued by patients.	Full attention: *"If a doctor is seeing you and he or she is on the computer or answering the phone, I think the doctor loses all humanity, they must pay attention to the patient. (…) that's where I consider whether the doctor is looking out for you." (P16).*<br><br>The patient talks and the professional listens: *"Listening to the patient is the least a professional can do. If you go to a professional, it is so that they listen to you, so that they can assist you correctly. (…) If they are a good professional, they are sure to listen to you. For the patient, being listened to means that they are interested in what has happened to you." (P27)*<br><br>The meaning of asking questions: *"It seems very important to me, to be asked how you are doing with your disease. It shows interest and concern and provides an indication of the type of professional you are." (P17)*<br><br>Professional commitment and attention to detail: *"It shows in the shifts. We can all do a job, but the attitude with which things are done is relevant. Maybe things don't work out but you put all your love and all your effort into it, so for me that's enough." (P2).*
Category: Personalized care<br><br>Describe the meaning of personalized care as narrated by patients.	Meaning of personalized care: *"It is important to note that the professional is interested in what you give importance to. That he or she knows you. Maybe knowing that your mother is sick, or your work." (P2),*<br><br>Professional considerations: *"I have concerns, I'm sure they won't solve all of them, but it is important to perceive that they are concerned about solving the most important ones for me." (P29). "If a patient goes to the doctor and says he has a problem, and the doctor says it's nothing, it doesn't help the patient. You will leave discouraged because the doctor didn't believe you." (P27). "It is a mistake to remove priests from hospitals because they help to provide support, a spiritual encouragement that gives strength. The fact that professionals have enormous respect for the moral values, human values, and beliefs of their patients, gives security. And that helps the patient a lot." (P15). "When they give information to a patient, they don't have to limit themselves to just providing information. You have to consider that this person has an emotional component (...) If you don't consider the emotional component that this person has, you may be directly undermining the recovery process that the person may have". (P1). "You are dealing with people who have their feelings, their*

	emotions and you mark them (...) You don't know what the person feels. You have to ask them." (P2). "Professionals should understand that stroke not only has physical consequences, but also emotional ones. They should be able to put themselves in the situation, understanding a little of what goes through your head when you have a stroke." (P20)<br><br>Share time and space with patients: *"I like it when they can spend some time and chat. That excites me (...) that he takes the time to listen to me, to sit down with me and to see what is good for me and to involve me." (P9) "The staff becomes a robot, and you become a number. They keep to their schedule, and we patients do what they tell us to do. It's the easy way out."(P29)*<br><br>Indicator of professional interest: *"The more time he spends with you, the more I think he is really interested in my case. Otherwise, he wouldn't stay even 4 minutes."(P20)*
Category: The professional's heart<br><br>These are those actions that for patients mean that the professional does not only apply their knowledge and skills, but above all their heart when treating them.	Professional "affection": *"The sparkle is giving a lot of love and affection (...) When you put your affection into something, everything feels better. It's something you notice in people, and it makes the difference between professionals." (P2). "There are professionals who are like a fine-tuned machine, who do their job perfectly. But there is no warmth in their manner... One must feel that one is not in the hands of a machine." (P10),*<br><br>A close professional: *"With what I have, I didn't need to be sugarcoated. But support was something else. Having a reference figure is very important, because you have another support to count on that you might not have expected, and they are there telling you <count on me>." (P1)*<br><br>Physical contact of the professional: *"When I arrived, just shaking hands was everything for me. I felt security, companionship. I knew that whatever I was going to wear was going to suit me."* (P18). *"Do you know what it's like to have someone give you a hug in this situation? It's what you need at that moment, a help you don't expect, the gesture that you're not alone." (P29)*<br><br>Smiling at the patient: *"The smile of the doctor and the nurse helps you a lot (...) when I go to see someone and they smile at me, they have already won me over. Every time I go to my doctor and they smile at me, I feel that I am a little cured. I don't need anything else." (P23)*
Category: Building a bond with the patient<br>This category describes those elements that help to build a bond with the professional.	The professional shares personal matters: *"Many times [the professional] would tell me his things, his problems, and at those moments I could put myself at his level and say something to help him, encourage him or give him advice." (P14). "Over time, a friendship develops. This even changes the way you talk about the treatment. At the end you ask him about his weekend, his family, as if you were a friend you meet on the street.*

	That's important." (P18)<br><br>Asking questions and expressing yourself without filters: *"I like it when they pay attention to me when they approach me like family. And since it's your family, you already loosen up and tell them what you think without problems." (P7)*<br><br>Putting yourself in the other person's shoes: *"I noticed that I didn't understand it. It seems like an everyday thing that does not require any effort, but for me it was a whole new world. It is necessary for a professional to understand what a stroke means and the consequences it has, from the patient's point of view (...) If they do, when I say I am tired, they understand that I am tired, because they are putting themselves in my place and know what is happening to me. That gives me confidence. Respecting others is much easier, when you put yourself in their place, and by understanding what happens to patients, everything is much easier." (P20)*<br><br>Trust is everything: *"Trust, it doesn't come because you're a doctor and you have to be trusted. You have to earn it through actions." (P6). "If there's no interest from the doctor and he doesn't care what I do, I'm not going to trust his judgment. If there's no interest, when I have something major, I'm not going to trust him to care." (P26)*<br><br>Loss of credibility: *"They didn't know, they had no idea. They are the doctors, and they didn't know." (P3). "What interest can they have in me getting better? I don't trust someone who has an obvious conflict of interest in everything they say about me. And the fact that they earn more money the worse off I am, so they have no interest in me getting well." (P9)*<br><br>Humor: *"Handling things with a sense of humor, it makes everything much more relaxed. When I met a professional who laughed and made jokes, it helped me. The feeling is different, it takes the drama out of everything." (P20)*<br><br>Camaraderie and complicity: *"It's essential. If you laugh with someone then it' s different (...) There is a camaraderie. And when you go to therapy, you make jokes, they encourage you, and the treatment is experienced differently."(P6). "For me, laughing is great. I found complicity in these assistants, it made me feel great. When the nursing assistant showered me, we laughed a lot, because it was like a very pornographic image. I would say, take some pictures of me, I'm going to send them to my colleagues, and we wouldn't stop laughing." (P9)*

Theme 2: Facilitating communication	
Categories	Narratives
Category: The patient as the recipient<br><br>This category describes those elements of the message that make it easier for the patient to understand the information.	Tailoring the message: *"You can tell when there is no closeness with the professional, because they only use technical terms, such as ischemic damage. And when you ask him what it is, he answers you with more words that you don't understand." (P6). "They are very technical, distant, there is no closeness, it's downright coldness." (P15).*<br><br>Speaking on the same level: *"The doctor who explains things and makes you understand, for me that' s a good professional. (...) They speak in another language, they see it as normal, but you are at another level, your language is much more limited, you don't understand what they want to tell you (...) the fact that we both speak the same language is crucial. For me it means speaking at the same level. The doctor speaks to you at a level that you can understand him even if you don't know the vocabulary he uses". (P6)*<br><br>Selecting another person to receive the information*: "They tell everything to my wife or my mother. When they don't tell me anything, it makes me angry. It bothers me that they tell things to my wife before they tell me." (P8).*<br><br>Acceptance of information: *"If the information is given to you by someone who half knows you, you don't take what they tell you the same way. You go away more convinced that what they tell you is correct and that what they have done to you is right. You don't argue with the information you are given." (P26).*
Category: The content of the message and the channel.<br><br>This category describes the content of the message that patients need.	Content of the information: *"Knowing how this disease works, allows you to regain some control of your life, and move forward, first with help and then on your own, step by step". (P6)*<br><br>Use of words of encouragement and support: *"When it comes to stroke, the professional has to be positive, to help you, to encourage you. Saying a positive word to you doesn't hurt. By encouraging me, he showed me that he understood perfectly well what I had, and I was trying hard to get better." (P17)*<br><br>Alternative channels of communication: *"Now there are a lot of telephone consultations, or through e-mails that they send you. All those things are fine, but you lose the proximity, the touch, the contact. I like to have another person in front of me, to see them, to see what their reactions are when I speak." (P23)*
Category: The professional as the conveyer.	Training in communication: *"First are the technical issues, your training as a professional. But you can be a*

This category describes aspects that the professional should include when communicating or conveying information to the patient.	*good technician, and a bad communicator with the patient." (P6). "It should not be an option; it would have to be included in their training. Teaching them in what to say, how to say it, how to interact with the patient when explaining things..." (P26).*<br><br>Avoiding bluntness during communication: *"It's not just about explaining things, you have to know how to explain it. When you have a certain responsibility, you have to know how to communicate. You have to give feedback in a non-aggressive way, even if it is negative feedback. You have to know how to give negative news without crushing anyone."(P29). "There are many ways to explain things to people. You have to tell the truth, not deceive them, but not traumatize them for life, and let them sink and shoot themselves." (P26)*<br><br>Focusing on losses: *"If they [the professionals] tell you that you are not going to recover any more, and they take their time to explain how far you are going to be able to go, it is as if they wanted to take away a goal to fight for. They should be careful with their words and evaluate how far they can go because of the implications that their words can have (...) There is a way of saying things that can directly destroy the progress that a patient can make, or the hope of that progress. A doctor can shatter you because what he says has an impact on you, it marks you. I keep pushing forward, but I am already marked by what you have told me." (P1)*
Theme 3: Promoting participation	
Categories	Narratives
Category: Barriers to participation. Barriers that patients experience when participating in decision making are described.	"I prefer not to express my opinion." *"When you don't know something, you have no choice but to put yourself in the hands of professionals. And since we are patients, you have to put up with it and do what they tell you." (P13)*<br><br>Last link in the chain: *"I had no say because I didn't know what was happening to me, or why it was happening to me, or what the consequences were. We are the last link in the chain. I couldn't have an opinion or act in any way." (P13).*<br><br>Professional barrier: *"It's not that they're worse professionals, but they have that top-down approach. Since I'm the one who knows, I'm going to tell you what to do." (P9). "The roles are very different. There is an invisible barrier that you notice right away; those who are in charge and those who are not in charge, those who know and those who don't know." (P10).*

	In the hands of others: *"I felt like I was in other people's hands. I have always been independent, and I was not comfortable with the feeling that my fate was in other people's hands and not mine." (P9). "You are a sick person. You are in a weak, submissive position. You're not in charge, you go to bed and get up when you're told. You don't play a role. You have no decision-making power. Nothing depends on you, I'm the last link." (P29).*
Category: Incentives to participate.<br><br>This category describes the stimuli that favor participation in patient decision making.	Professional actions to avoid: *"It makes me angry that they change things and don't tell me the reason. They have made decisions that I don't think are bad either, but they have to tell me. (P8). "I agree that they should evaluate you, and teach others, but they shouldn't go to a corner of the room and start talking among themselves, as if you weren't there." (P29).*<br><br>Being consulted/stimulating participation: *"There are two different approaches from health care providers. There are those who don't allow you to participate and tell you what to do, and there are those who consult you. I like the people who involve you more. It means that they ask me what I expect, how I want to do it. It has taken me into consideration as a person who can contribute things. It's talking together to see how we can do it." (P9)*<br><br>Teamwork: *"Recovery is 50% from the doctor who is treating you, and the other 50% is from the person they are treating." (P6). "I have felt very good in that aspect, because they have let me be the one to set the limit of my possibilities. It's not letting me do whatever I want, but they have always acted respecting my decisions." (P6).*<br><br>Redirecting the patient: *"People [professionals] listen to you, and then they tell you that you have to improve. They teach you the faults you have. They teach you and you get better. That's getting involved." (P8)*

Table S2. Facilitators/enablers and Barriers of professional support, communication and participation with stroke patients.

	Facilitators/enablers	Barriers
Supporting the stroke patient*	• Personalized care<br>• Considering individual preferences, beliefs and/or spiritual needs.<br>• Proximity, warm treatment, establishment of physical contact, patient support.<br>• Sharing time and space<br>• Getting to know the professional and exchanging personal experiences<br>• Considering the patient's emotions and put yourself in their shoes<br>• Sense of humor and welcoming with a sincere smile.	• Standard and fast automated processing<br>• Making errors or mistakes in treatment<br>• Failure to recognize mistakes<br>• Receiving the same information from different professionals<br>• Lack of knowledge of the disease or its evolution<br>• When the patient speaks and the professional does not show signs of listening (keeps writing, doesn't look into the patient's eyes).<br>• Feeling that the professional is not interested in your narrative.
Communication with the stroke patient*	• Tailoring information to the patient's ability<br>• Facilitating means of communication<br>• Optimal information is that which provides data on the disease, its causes, implications for life, consequences and possible evolution.<br>• Give the patient a choice, to whom the information is passed on to	• Use of technical language<br>• Not giving information or delaying it to avoid worrying the patient.<br>• Choosing to give information to the family without confirming with the patient<br>• Not being able to consult with a professional, or not being answered (e.g., email consultations)

	• Including words of encouragement and motivation<br>• Information about the future reduces uncertainty<br>• Face-to-face consultations<br>• Specific training of professionals in communicating and providing information	• Focusing on losses and limitations<br>• The way of conveying information - very direct-no filters"<br>• Avoiding leaving the relationship with the patient to ICTs<br>• Avoiding redirecting or teaching a patient after a failure
Participation of the stroke patient*	• Asking the patient about their degree of participation in decision making<br>• Provide information and reasons for changes in treatment<br>• Asking which therapeutic itinerary the patient wishes to follow<br>• Asking whether the prescribed treatment may cause any problems or difficulties in the patient's life<br>• Demonstrating interest and involvement in patient participation<br>• Respect the patient's limits and opinion	• Leaving the patient out of plans and activities<br>• Feeling that they have no say, no action, the professional is in charge<br>• The doctor's attitude that he/she knows more about how the patient is experiencing the disease than the patient himself/herself.<br>• The doctor’s know-all attitude, just because he/she is a doctor<br>• Avoiding asking or discouraging the patient to give feedback on any aspect of the treatment or care received

*Procedure: After identifying the final themes, the research team reviewed the codes and categories, and constructed a matrix where the identified barriers and facilitators for Supporting the stroke patient, Communication with the stroke patient, and Participation of the stroke patient were distributed.
